# Clinical Peptidomics: Advances in Instrumentation, Analyses, and Applications

**DOI:** 10.34133/bmef.0019

**Published:** 2023-05-15

**Authors:** Lin Li, Jing Wu, Christopher J. Lyon, Li Jiang, Tony Y. Hu

**Affiliations:** ^1^ Center for Cellular and Molecular Diagnostics, Department of Biochemistry and Molecular Biology, School of Medicine, Tulane University, New Orleans, LA, USA.; ^2^ Department of Laboratory Medicine and Sichuan Provincial Key Laboratory for Human Disease Gene Study, Sichuan Academy of Medical Sciences and Sichuan Provincial People’s Hospital, Chengdu, China.; ^3^ Department of Clinical Laboratory, Third Central Hospital of Tianjin, Tianjin Institute of Hepatobiliary Disease, Tianjin Key Laboratory of Artificial Cell, Artificial Cell Engineering Technology Research Center of Public Health Ministry, Tianjin, China.; ^4^ Department of Biomedical Engineering, School of Science and Engineering, Tulane University, New Orleans, LA, USA.

## Abstract

Extensive effort has been devoted to the discovery, development, and validation of biomarkers for early disease diagnosis and prognosis as well as rapid evaluation of the response to therapeutic interventions. Genomic and transcriptomic profiling are well-established means to identify disease-associated biomarkers. However, analysis of disease-associated peptidomes can also identify novel peptide biomarkers or signatures that provide sensitive and specific diagnostic and prognostic information for specific malignant, chronic, and infectious diseases. Growing evidence also suggests that peptidomic changes in liquid biopsies may more effectively detect changes in disease pathophysiology than other molecular methods. Knowledge gained from peptide-based diagnostic, therapeutic, and imaging approaches has led to promising new theranostic applications that can increase their bioavailability in target tissues at reduced doses to decrease side effects and improve treatment responses. However, despite major advances, multiple factors can still affect the utility of peptidomic data. This review summarizes several remaining challenges that affect peptide biomarker discovery and their use as diagnostics, with a focus on technological advances that can improve the detection, identification, and monitoring of peptide biomarkers for personalized medicine.

## Introduction

Multiple “omics” approaches (e.g., epigenomics, genomics, transcriptomics, proteomics, and metabolomics) have been employed in biomarker discovery studies, attempting to identify new markers to improve disease diagnosis and treatment and thereby improve patient survival rates and outcomes. This research has contributed to a better understanding of disease pathophysiology, offered new opportunities for diagnosis and prognosis, and led to improved patient management. For example, these studies have yielded breakthroughs in the early diagnosis, prediction, and prognostic evaluation of several cancers [1] and other diseases or conditions, including stroke [2], chronic obstructive pulmonary disease [3], and Alzheimer’s disease (AD) [4].

Effective biomarker discovery studies should identify factors that provide real-time information that accurately and sensitively reflects a specific pathophysiologic state. Small molecules, including peptides, are ideal targets for such markers because they can often readily cross barriers (e.g., the vascular wall and blood brain barrier) that attenuate the accumulation of factors produced by tissues of interest in biofluids that are sampled for disease diagnosis. Peptides are of particular interest because they can exhibit properties useful in both diagnostic and therapeutic applications [5,6]. These include the potential to exhibit cell/tissue-specific secretion, cross cell membranes or tissues, be produced by cell/tissue/disease-specific enzyme activities, or interact with factors expressed on specific cells or tissues.

The term peptidomics, first introduced in the 2000 Association of Biomolecular Resource Facilities conference “From Singular to Global Analyses of Biological Systems,” refers to the study and quantification of endogenous peptides of 2 to 50 amino acids (0.2 to 10 kDa) [7]. Such peptides are found in all cells and biofluids and play vital roles in specific physiological and biological functions. This includes peptides that influence vasodilation [8–10], oxidative stress [11], cell differentiation [12,13], apoptosis [14], or exhibit antimicrobial activity [15], as all these processes can influence disease progression. Several of these responses are regulated by peptides that serve as cytokines, chemokines, growth factors, peptide hormones, or inflammatory agents. Changes in specific peptides or peptide patterns may thus serve as potential biomarkers of normal physiologic or disease-specific pathologic processes or pharmacologic responses to therapeutic intervention.

**Fig. 1.**
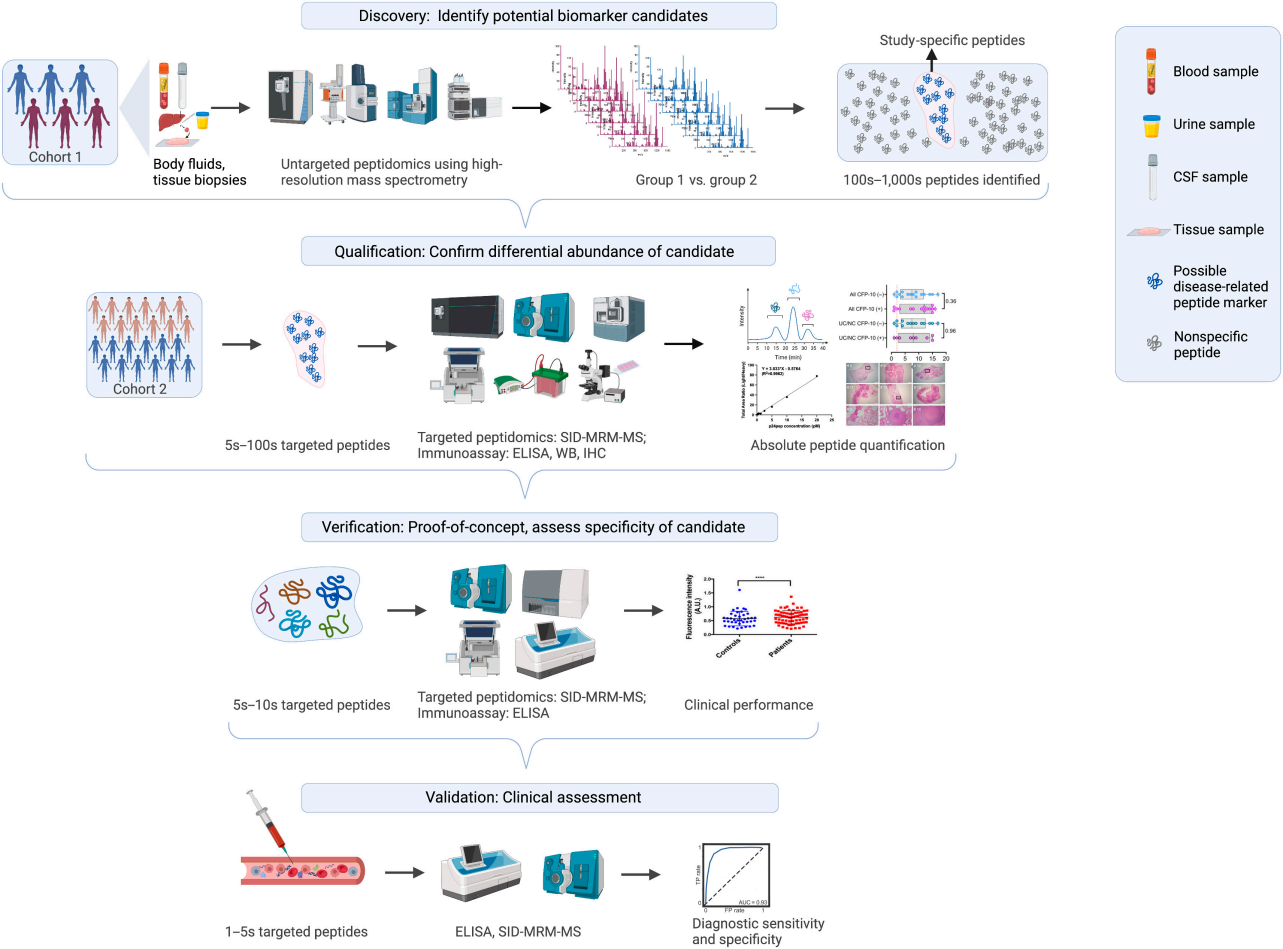
Standard 4-stage MS workflow used for peptide biomarker discovery and validation. An untargeted MS analysis is used to identify peptide expression differences between a disease and a disease control group (discovery). Targeted MS is then used to confirm the differential expression of a subset of these peptides in an independent study cohort (qualification), to evaluate the specificity of a subset of these qualified peptides for the disease of interest versus other conditions with similar symptom profiles (verification), and to characterize their diagnostic performance in appropriate study populations (validation). WB, Western blot; IHC, immunohistochemistry; A.U., arbitrary units; TP, true-positive; FP, false-positive; AUC, area under the curve ; SID, stable isotope dilution. ****P < 0.0001.

Peptidomic approaches represent a powerful means to identify new peptide biomarkers for diagnostic and therapeutic applications, but such biomarkers must meet multiple criteria at discovery and validation before they can be employed in clinical applications (Fig. 1) [16]. This development process can be broken down into 4 major stages: discovery, which identifies potential biomarker candidates; qualification, which verifies their expression in the targeted disease state; verification, which evaluates their specificity for the targeted disease versus other conditions that may produce similar symptoms; and validation, which analyzes their diagnostic performance in appropriate study populations [17]. Peptidomic biomarker discovery studies typically use untargeted analysis or unbiased screening methods that employ mass spectrometry (MS) to analyze patient specimens to identify peptide differences that can distinguish individuals with and without a specific disease [18]. Such analyses commonly use specimens obtained from closely matched individuals who have a specific disease or who do not have this disease but present with similar symptoms.

Over the past 3 decades, MS systems that use a variety of separation and ionization methods have emerged as essential platforms for rapid and high-throughput peptide identification and characterization [19]. Their successful use in biomarker studies, however, requires the use of a robust and reproducible analysis approach that is coupled with a refined computational framework [20]. Such analyses must also address sensitivity and specificity concerns because biomarker peptide detections can be substantially affected by the matrix complexity of the analyzed specimen as it is more challenging to measure minor peptide changes against the background arising from complex mixtures of high-abundance proteins [21]. Effective peptide extraction methods must therefore be coupled with sensitive analytical approaches to effectively detect and characterize perturbations in peptide content in most specimen types [22]. Selected biomarker targets must then be verified and validated in appropriate secondary studies. Finally, validated peptide biomarkers can then be transferred to high-throughput assays suitable for use in clinical applications, such as fully automated chemiluminescent immunoassays, or serve as targets for imaging approaches that detect disease lesions by the localized enrichment of nanoparticle (NP) affinity probes.

This review will focus on challenges and advances in peptidomic analyses, describe key steps in such analyses, and discuss the potential utility of specific peptides as disease biomarkers and approaches that may have clinical utility for such analyses. We will summarize potential applications for different analytical methods, including the use of MS methods in peptide biomarker discovery and validation studies; nanopore approaches to detect and identify specific peptide biomarkers; immunoassay-coupled approaches that can be employed in high-throughput assays; and NP-based assays that use synthetic probes to detect disease-associated enzyme activity changes in situ. We will also discuss studies completed in the past decade that have used peptide biomarkers and reported outcomes.

## Peptidomics Analysis: Challenges and Advances

Despite intense interest and investment in peptidomics for biomarker discovery, few peptide biomarkers are now used in clinical practice (Table), reflecting the numerous remaining issues associated with peptide biomarker analyses, including challenges involved with sample preparation (peptide extraction and enrichment/purification) and data analysis (accurate detection and identification with appropriate databases and software).

**Table.** Example of important peptide biomarkers used in clinical diagnoses.

**Peptide** *	**Molecular mass (kDa)**	**Clinical diagnostic use**	**Reference**
NT-proBNP	8.6	Cardiovascular diseases	[23]
Insulin	5.8	Diabetes	[24]
Amyloid beta peptide	4.8	Alzheimer disease	[25]
Pro-GRP	26	Neuroendocrine tumor	[26]
PINP	14.2	Bone turnover	[27]
Calcitonin	3.4	Medullary thyroid carcinoma	[28]
C-peptide	3.1	Diabetes	[29]
Gastrin	2.1	Ulcers and diarrhea	[30]
Osteocalcin	5.8	Osteoporosis	[31]
Cystatin C	13.3	Renal failure	[32]
ANF	15	Heart failure	[33]

* NT-proBNP, N-terminal pro-B-type natriuretic peptide; Pro-GRP, pro-gastrin-releasing peptide; PINP, procollagen type I N-terminal propeptide; ANF, atrial natriuretic factor.

### Sample preparation

Peptidomic analyses have been performed on a diverse array of sample types, including biofluids (urine, plasma/serum, saliva, tears, and cerebral fluid), tissue biopsies, and primary cell isolates and cultures. Each of these specimen types has advantages and disadvantages that can determine its suitability for a particular analysis. The targeted peptidomic information must be detectable in a given specimen, but other factors apply if multiple sample types can meet this basic threshold. For example, biofluid specimens are often more easily obtained than tissue samples to facilitate serial analyses, including those intended to detect rapid physiologic changes, and may be less subject to sampling bias or processing effects than tissue biopsies or cell isolates. Furthermore, the homeostatic nature of some biofluids (e.g., plasma/serum and cerebral fluid) may improve the accuracy of repeat measurements designed to detect indicators of modest physiologic changes. Blood samples are frequently used in biomarker studies because of their ease of collection and utility in detecting patterns that can be used for targeted diagnosis of pathophysiologic states [34]. However, tissue biopsies and enriched cell samples can sometimes isolate specific tissue regions and cell types associated with a disease or a dysregulated physiologic state to increase their relative contribution to the proteome or peptidome derived from a biospecimen [18]. For example, one study analyzed the secretome of cartilage explant ex vivo cultures to identify endogenous peptides differentially secreted by cartilage from osteoarthritic versus healthy tissue biopsies that could serve as candidate peptide biomarkers for osteoarthritis [35].

**Fig. 2.**
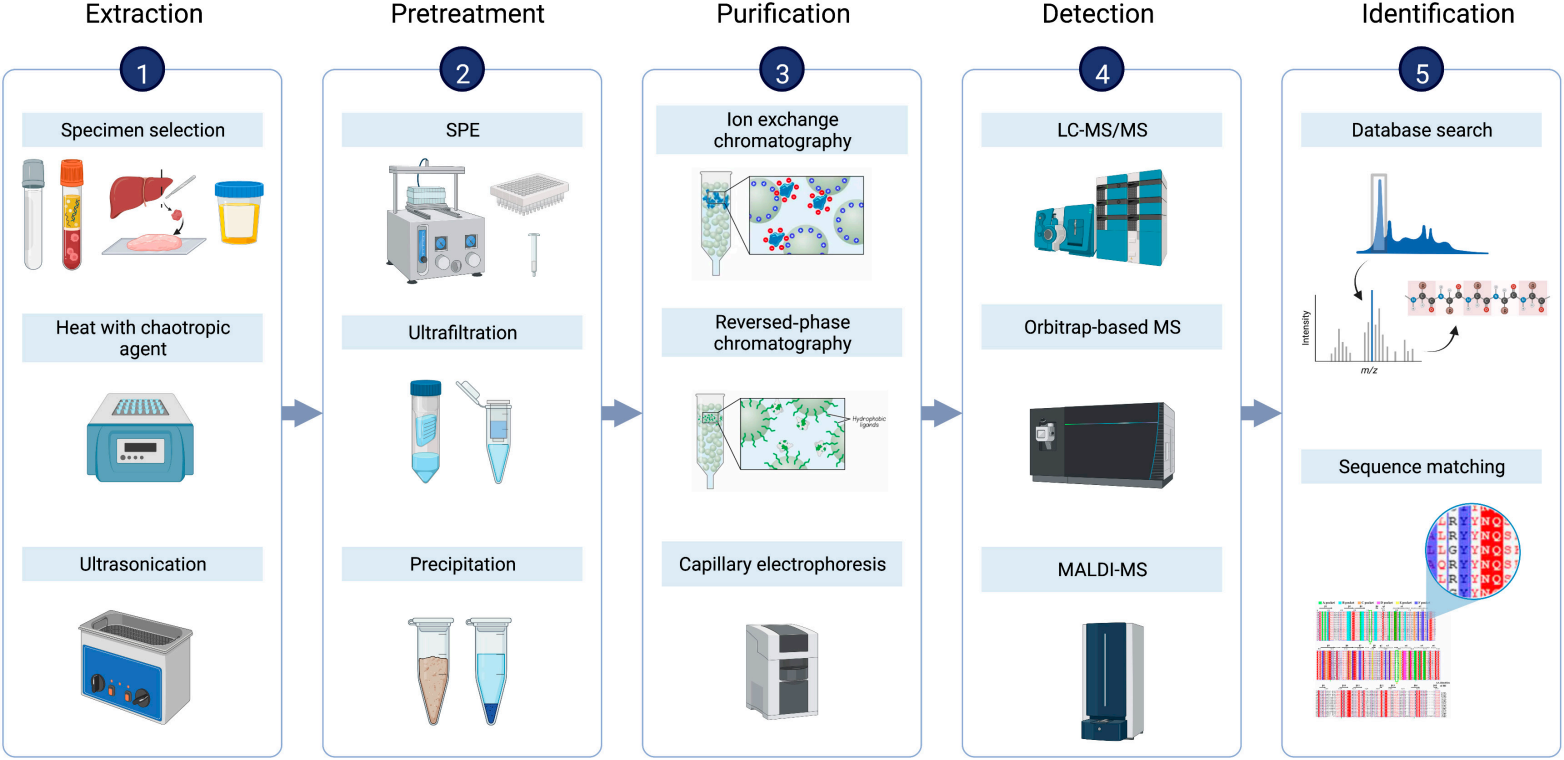
Schematic of key steps in standard peptidomic workflows. Specimen types selected for analysis are first chosen to maximize the odds of detecting peptide patterns of interest and then extracted and processed to liberate these peptides from potential interaction partners and protect them from degradation/modification by endogenous enzyme activities by adding protease inhibitors or protein denaturation procedures. Samples are then usually subjected to size exclusion or selective precipitation to enrich peptides from larger proteins and protein fragments, and isolated peptides are then fractionated by their inherent properties, characterized by MS and identified by bioinformatics. SPE, solid-phase extraction.

Standardized sample preparation protocols are critical to ensure efficient and reproducible peptide recovery, but those that employ multiple pretreatment steps to maximize peptide recovery may have detrimental effects (Fig. 2). For example, sample processing time may increase peptide losses and variability, because peptides are highly susceptible to degradation in many biological matrixes. It is therefore essential to minimize endogenous protease/peptidase activity during sample collection and preparation. Chaotropic agents or specific and/or broad spectrum protease inhibitors are often added to samples before extraction or storage to reduce proteolysis. Rapid sample freezing or heat inactivation steps are often employed for the same purpose.

Common diagnostic sample components may also inhibit MS detection of low-concentration target peptides because of the “masking” effects of lipids, salts, and peptides derived from highly abundant proteins (e.g., albumin; present at a 40 to 50 g/l in serum) to suppress the detection of target peptide ions. Different sample pretreatment methods have thus been developed to capture, partition, fractionate, or enrich peptides and/or deplete masking factors to improve peptide detection. For untargeted analyses, these include selective enrichment via acid- or solvent-mediated protein precipitation, solid-phase extraction, or ultrafiltration or molecular weight cutoff approaches, which can produce 100× to 1,000× enrichments in some cases [36,37].

Biospecimens are often pretreated with detergents, solvents, acids, or chaotropic agents to selectively denature and precipitate undesired proteins before analysis. For example, serum/plasma samples are frequently fractionated by adding a 2× volume of acetonitrile to selectively denature and precipitate large and abundant proteins in these specimens, while allowing small proteins and peptides to remain in solution. Zwitterions, small molecules that contain separated regions of positive and negative charge (e.g., taurine), are sometimes also added to solvent-mediated precipitation procedures to disassociate peptides and small proteins from highly abundant proteins to enhance their recovery. However, selective precipitation can also cause marked depletion of desired proteins without fully depleting abundant proteins that may have masking effects.

Centrifugal ultrafiltration, the most widely used means to extract peptides from higher-molecular-weight proteins, employs centrifugal force to selectively drive low-molecular-weight molecules through a semipermeable size exclusion membrane. MS analyses of serum samples processed by this approach have identified >300 unique peptides with <2 ppm of mass accuracy [38]. Nanoporous materials used in solid-phase enrichment methods can also be engineered to optimize their physicochemical properties (pore size, pore structure, and surface affinity) to selectively enrich low-molecular-weight and low-abundance protein and peptides [39], including those with specific properties. For example, surfaces modified with an 18-carbon (C18) chain can selectively enrich nonpolar peptides, those modified with C2–C8 chains can enrich less-hydrophobic peptides, and anionic and cationic modifications can enrich positively and negatively charged peptides, respectively [39]. In these approaches, the composition of the solvent phase is used to regulate the selectivity of peptide binding to and elution from a surface.

### Detection methods for peptide biomarkers

The development of multiple high-performance separation and analytical methods has streamlined the identification and quantification of new peptide biomarkers. Liquid chromatography (LC) separation methods (e.g., reversed phase, size exclusion, and cation/anion exchange) can resolve distinct peptide populations and are widely used for peptide fractionation before MS analysis. Peptide fractions generated by these methods are primarily analyzed at high resolution by matrix-assisted laser desorption/ionization time-of-flight (MALDI-TOF) MS or electrospray ionization (ESI)-MS. The former method analyzes evaporated fractions loaded at separate spots on a detection plate, while the latter analyzes liquid fractions as they elute from an LC gradient or as they are individually loaded onto ESI-MS systems. High-resolution Orbitrap, ion trap, and quadrupole time-of-flight instruments used in these analyses can differentiate peptides differing by a single mass unit with a 10-ppm error in samples that contain large and dynamic variations in peptide abundance and charge (charge states ≤ 10+). MS systems that employ ion traps to detect peptide fragments generated by a single fragmentation step (MS^2^) or multiple fragmentation steps (MS*^n^*) can increase detection selectivity, as these methods can monitor multiple fragments from a single peptide to determine its sequence identity [40].

Peptide ion separations can also be enhanced by introducing ion mobility (IM) separation between the ionization and mass/charge separation steps of an MS analysis, either as a standalone technique or as a precursor to peptide fragment separations. The arrival time of an ion at the end of the IM separation path increases with the ion’s mass and collisional cross section (CCS), with the gas phase used in this procedure, so that the CCS value of each ion reflects its rotationally averaged cross-sectional area. Ions with smaller CCS values traverse the IM separation path faster than those with larger CCS values, adding another separation dimension beyond mass and charge to ion identifications [41]. For example, IM techniques (e.g., differential mobility spectrometry and trapped IM spectrometry) have been employed to identify and monitor isomeric peptides and improve the signal-to-noise ratio of targeted analytes without MS^2^ or MS*^n^* analyses [42,43].

### Data analysis

Peptidomic analyses require knowledge of the peptides produced from precursor proteins by endogenous protease activities. However, accurate identification of such peptides remains challenging, even with efficient separation strategies, because of the diverse array of protease activities that can influence their production. Conventional proteomic analyses typically use a single exogenous protease to produce peptides that have N or C termini that are determined by this protease alone. Peptides produced by the combined activity of endoprotease and exoprotease activities in biospecimens lack this homogeneity and are thus difficult to identify by widely used bioinformatics tools designed to analyze MS datasets that contain peptides produced by a single protease activity. This can produce false-positive detection rates 100-fold greater than observed in single-enzyme proteomic studies.

Databases used to identify endogenously produced peptides thus need to account for a range of protease activities and other posttranslational modifications (PTMs) that can influence peptide identifications [44]. For example, acetylation or pyroglutamylation events can alter the net charge states of some peptides to prevent their identification by approaches that use positive mode analysis [45]. However, it is important to capture and analyze this PTM information, when possible, and a major advantage of peptidomic workflows is their ability to detect degradation products, sequence variants, and combinations of PTMs that may not be detectable by other approaches [46].

New computational approaches, including those based on machine learning and data mining methods are under continuous development as support tools to aid in the identification and annotation of detected peptides and their source proteins. Web-based databases containing peptides were first reported in 1998 [47]. These can be useful in specific cases, but comprehensive and integrated peptide-focused databases are not easily found and require a background in bioinformatics to use, reducing their utility to users who lack advanced programming skills. Most databases developed specifically for peptide analyses, including PepBank and PeptideAtlas, also require prior knowledge of the source(s) of the peptides of interest. Notably, the recently developed Peptipedia database, which employs a machine learning model to support biological activity classifications, includes a user-friendly interface to facilitate peptide sequence searches. This platform uses a NoSQL database system that contains >92,055 registered and described peptides and is the most extensive peptide sequence database annotated with peptide activity information that has been reported to date [48].

Native database search tools or de novo peptide sequencing packages are required to compare experimental tandem MS (MS/MS) ion spectra with theoretical spectra predicted for each database peptide or an array of all possible peptides to identify the specific peptides present in a specimen, respectively. Database search tools are simple to use and limit the scope of the search and are thus often preferred for analysis. Representative database search tools include MSFragger [49], Mascot [50], SEQUEST [51], and MaxQuant [52], which use different search algorithms. No database information is required for de novo sequencing packages, as these programs compute the best possible match for all possible amino acid combination for peptide ions in experimental MS/MS spectra, but this approach requires more computational power, is slower, and requires the use of MS/MS spectra with high mass accuracy. Representative de novo sequencing packages include PEAKS [53], PepNovo [54], NovoHMM [55], and Lutefisk. PEAKS is commonly used for de novo sequencing studies because of its superior sensitivity and positive predictive value [56]. PEAKS DB software was developed by combing the de novo algorithm and the database search approach, to further improve sensitivity and accuracy for peptide identification [57].

## Analytical Methods for Peptide Analysis

In recent decades, analytical proteomic/peptidomic methods have become faster, more refined, and less expensive to increase their potential for application in personalized medicine, where sensitive assays are needed to evaluate treatment responses and to guide informed strategies to improve therapeutic interventions. MS remains the primary tool for the discovery and analysis of peptide biomarkers, as it can reproducibly separate and quantify diverse peptides in complex samples based on small differences in their mass-to-charge (*m*/*z*) ratios as they pass through an electromagnetic field [58]. Numerous untargeted analyses performed using capillary electrophoresis-MS (CE-MS), LC-MS, and MALDI-TOF MS systems have demonstrated their ability to identify and reproducibly detect peptide biomarkers [59]. Nanopore-based approaches may also have utility for the detection of peptide biomarkers but cannot currently detect such markers directly from complex biological matrixes and, thus, are not suitable for biomarker discovery studies [60].

Biomarker discovery studies employ untargeted peptidomic analyses and statistical analysis of the resulting dataset to identify potential biomarker candidates, after which targeted analyses and validation studies are performed to verify detected markers [16]. Several techniques can be used to detect target biomarkers in clinical applications after their validation, including MS methods that use selective or multiple reaction monitoring (SRM or MRM) for verification and validation, immunoassays, and nanopore assays [59]. Because of the complex and variable proteomic nature of many diagnostic specimens, the conditions required for optimum sensitivity and to accurately quantify a target peptide are often determined using samples obtained from healthy subjects that have been spiked with synthetic reference proteins. The applications and limits of the most common techniques for peptide detection and quantification are discussed below.

### CE-MS-based peptide detection and quantification

CE-MS couples CE, which separates molecules by mass and charge state as they transit a capillary in response to electroosmotic force [61], with on-line MS analysis to provide rapid and high-resolution separations and molecular mass identifications. CE can be considered an orthogonal and complementary separation method to LC-MS given the low overlap in peptides detected by both methods (30%) in a comparison study [62]. This study found that fewer peptides were uniquely detected by CE-MS than LC-MS (20% versus 50% of total peptides), likely because of the greater loaded sample mass and longer separation times possible with LC-MS analyses. CE-MS/MS and LC-MS/MS are thus highly complementary, and employing both can substantially increase sequence coverage. However, CE still has potential advantages when used alone, including its small fraction volumes and its ability to run offline with MALDI-TOF MS [63] or online with triple quadrupole MS, and CE-MS has been increasingly used in peptide biomarker analysis in the past decade. For example, one recent study used CE-MS to establish a cholangiocarcinoma (CC)-specific support vector machine-based peptide marker model for CC diagnosis [64] to identify urine peptide biomarkers that revealed altered distribution between patients with and without CC. Another study analyzed CE-MS urine data from individuals with different solid tumor types (bladder, prostate, and pancreatic cancers, as well as renal cell carcinoma and CC) to identify cancer-associated peptide markers linked to general systemic effects during cancer progression [65]. A third study identified 273 urine peptides that substantially differed between patients with chronic kidney disease (CKD) of different etiologies and healthy controls and combined these into one classifier, CKD273, which was evaluated for CKD diagnosis accuracy [66].

### LC-MS-based peptide detection and quantification

After the development of ESI technology [67], LC-MS became the method of choice for high-throughput peptide detection, as it permits simultaneous high-resolution separation and sensitive detection to facilitate the rapid discovery and validation of peptide biomarkers. LC-MS has been used to identify peptide biomarkers for pancreatic neuroendocrine tumors [68], primary immunodeficiency disorders (PIDD) [69], heart failure (HF) [70], and schizophrenia [71]. One group analyzed the plasma peptidomes of patients with neuroendocrine pancreatic tumors by using organic solvent extraction and nano- and high-flow LC-MS to identify several peptides that differ between glucagonoma, insulinoma, and control samples [68]. These included peptides derived from proglucagon, chromogranin A or B, and other peptide hormones and proteins associated with the regulation of peptide secretion. Another group studying PIDD used a high-throughput LC-MS^2^ proteomic screening approach to simultaneously identify peptides derived from 3 proteins: the transmembrane protein cluster of differentiation 3 (CD3) and the intracellular proteins Wiskott–Aldrich syndrome protein (WASP) and Bruton’s tyrosine kinase (BTK), as their absence was found to accurately identify patients with PIDD [69]. A third group investigating HF employed an immunoaffinity-based LC-MS^2^ assay to measure an N-terminal pro-B-type natriuretic peptide (NT-proBNP)-specific tryptic fragment that detected a marked serum NT-proBNP increase in rats treated to induce cardiac hypertrophy, corroborating the relationship between serum NT-proBNP and cardiac hypertrophy [70]. Finally, LC-MS^2^ and a label-free ion quantification method based on data-dependent acquisition were used to identify peptides altered in the postmortem brain tissue of patients with and without schizophrenia [71]. Notably, one of the identified peptides (pepH) was determined to influence cell viability, suggesting that it and perhaps other peptides, produced by altered protease activity, could serve as novel targets for schizophrenia and potentially other neuropsychiatric diseases [71].

### MALDI-TOF MS-based peptide detection, quantification, and imaging

MALDI-TOF MS is a rapid and highly reliable means to detect specific proteins and peptides and has several advantages versus other methods [72]. These include its speed, low per sample cost, and high tolerance to interferents present in many clinical specimens, including salt. This permits it to determine comprehensive “fingerprints” of peptides derived from proteins present in biological fluids to identify disease-associated biomarkers. Furthermore, this tolerance to sample interferents frequently allows the use of weak cation exchange magnetic beads (WCX-MBs) to directly isolate peptides from clinical specimens for immediate MALDI-TOF MS analysis. In the past decade, MALDI-TOF MS has been used to diagnose hepatocellular carcinoma bone metastases [73], oral squamous cell carcinoma (OSCC) [74], malignant pleural effusion of lung cancer [75], and *Leishmania donovani* infection [76]. WCX-MB-coupled MALD-TOF MS, and a neural network model was employed to identify serum peptides that distinguished hepatocellular carcinoma cases with and without bones metastases and to build a model containing 6 peptides that could distinguish these cases with 85% sensitivity and 86% specificity [73]. A similar WCX-MB MALDI-TOF MS peptidomic analysis approach performed on saliva obtained from an OSCC cohort determined that increased expression of the salivary peptide histatin-3 correlated with OSCC progression [74]. In a lung cancer study that analyzed pleural effusion samples from patients with lung cancer and tuberculosis by WCX-MB MALDI-TOF MS, a 5-peptide signature was found to diagnose lung cancer with 94% sensitivity and 100% specificity and to outperform the diagnostic sensitivity of carcinoembryonic antigen [75]. Finally, a WCX-MB MALDI-TOF MS study that analyzed serum from mice infected with and without the parasite *L. donovani* identified serum peptide profiles that distinguished infected mice by day 3 postinfection with 92% sensitivity and 97% specificity [76].

MALDI-MS imaging (MALDI-MSI) can also be used to characterize peptide composition to provide a spatial molecular analysis that can be combined with conventional histological data to analyze the distribution of hundreds of targets in histologic regions of interest [77]. This offers an optimal means to identify specific biomarkers and explore the complexity and heterogeneity of malignant diseases, including pituitary tumor [78,79], chondrosarcoma [80], and neurodegenerative diseases [81,82]. Notably, these molecular profiles may highlight tumor heterogeneities that may distinguish cancer subtypes as well as low- and high-grade cancers to inform treatment decisions. For example, one MALDI-MSI study detected 427 peptide peaks that could be used to distinguish regions of intratumor heterogeneity in microscopically identical chondrosarcoma sections [80]. The ability to rapidly provide spatial distributions of molecular data (within 30 min of sample collection) may also permit near-real-time delineation of tumor boundaries, as indicated by a study that employed MALDI-MSI to detect and discriminate malignant and nonmalignant pituitary gland tissue [78]. MALDI-MSI is also increasingly used to detect differences in target neuropeptide distribution and abundance in central nervous system (CNS) tissue from individuals with and without neurodegenerative diseases, as the diagnostic and potential therapeutic values of these peptides are gaining recognition [83]. For example, a high-resolution MALDI-MSI method developed to evaluate changes in neuropeptide localization in the brains of a rat Parkinson’s disease (PD) model detected neuropeptide differences in tissue with and without PD lesions and in PD lesions in animal treated with and without L-DOPA [84]. MALDI-MSI localization of amyloid beta (Aβ) peptide deposits in AD patient brain tissue also found that Aβ42/Aβ43 and Aβ36 to Aβ41 peptides revealed selective deposition in senile plaques and leptomeningeal blood vessels [85], respectively, likely due to a difference in their aggregation rates.

### Nanopore-based peptide identification

**Fig. 3.**
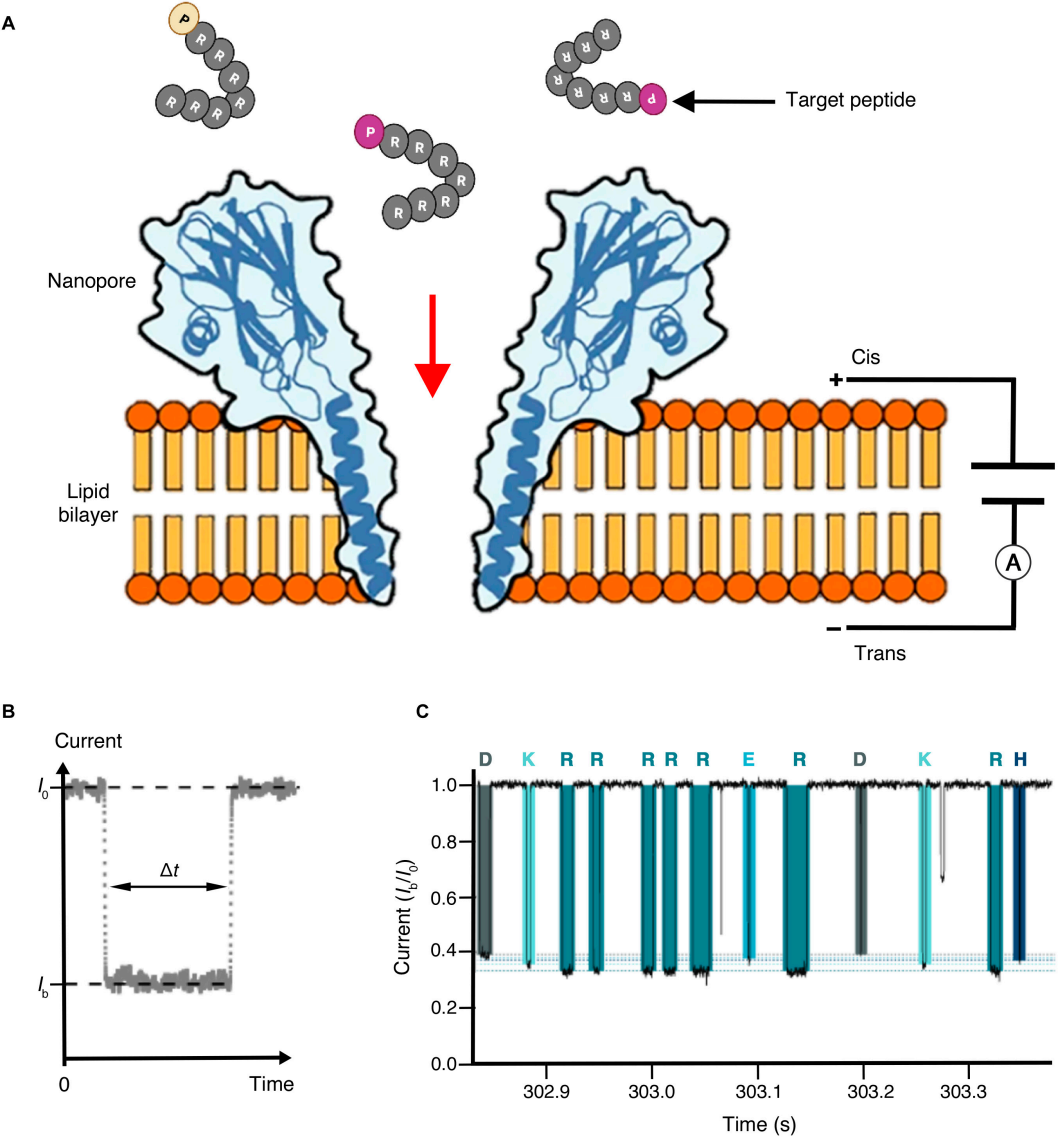
Nanopore detection of target peptides. (A) Schematic of a nanopore sensor device where peptide interaction with a protein nanopore in a lipid bilayer under an applied current is read to distinguish a target peptide. (B) Each target peptide present on the cis side of the bilayer can interact with the nanopore to induce a transient blockade of the ionic current, which is characterized by the mean residual current *I*_b_ and the blockade duration time Δ*t*. (C) Example trace readout for relative residual pore current (*I*_b_/*I*_0_), showing different current blockades for arginine heptamers ending with different amino acids, as indicated by single-letter amino acid codes above (adapted from [88]).

Scalable low-cost peptide analyzers are needed to meet the increasing demand for high-throughput peptidomic studies and personalized medicine. MS^2^ is the most useful means now available to identify and quantify target peptides with high precision in complex matrices, but MS^2^ systems are expensive, high maintenance, and require substantial infrastructure. Nanopore systems cannot distinguish minor peptide sequence changes with the precision of MS systems but permit rapid and inexpensive detection of low-abundance proteins and peptides from complex biological samples using portable devices suitable for use in resource limited settings. Nanopore sensors were first used for DNA sequencing as they can detect nucleotide-specific changes in ionic current as a single-strand DNA molecule transits a nanopore under a constant applied potential [86]. Subsequent advances in nanopore technology have allowed specific detection of DNA, RNA, protein, and peptide targets, with a 2012 study appearing to be the first to employ a nanopore system for peptide identification [87]. Similar to nucleic acid sequence analyses, it should also be possible to identify specific amino acids as their distinct physical properties may produce different ion current amplitudes and durations as they transit a nanopore (Fig. 3). However, this is more complicated in practice, and several major challenges remain for nanopore-based protein/peptide identification approaches.

First, unlike nucleic acids, proteins and peptides do not have uniform charges but instead exhibit wide variations in charge distribution that affect their ability to interact with and transit a nanopore in response to an applied electrophoretic force. Two major approaches have been used to overcome this issue. One method non-covalently or covalently modifies a polypeptide to, respectively, give it a uniform or directional charge that facilitates its interaction with and transition through a nanopore. Non-covalent methods typically use an ionic reagent, such as sodium dodecyl sulfate that uniformly interacts with a polypeptide sequence, while covalent modification methods frequently attach an oligonucleotide strand to the N or C terminus of a polypeptide. Polypeptide–nanopore interactions in the first method should primarily be determined by the N- and C-terminal distribution of charged resides and, potentially, residues whose size or conformation could hinder nanopore entry and passage. Such interactions may thus considerably vary from peptide to peptide and produce peptide signatures where the signal from one orientation dominates to a variable extent. Conversely, in the second approach, the N- or C-terminal modification acts as a highly charged lead sequence that drags the polypeptide through the nanopore in a single orientation. Solution pH can also play an important role in polypeptide–nanopore interactions, particularly for polypeptides not modified as above, by altering the charge state of side chains in the target polypeptide or nucleopore, especially for amino acids at the nanopore constriction [89].

A second major issue is that proteins and some peptides form stable secondary structures that must be disrupted for them to thread through a nanopore. This can be accomplished by the addition of strong chemical denaturants such as sodium dodecyl sulfate or guanidine hydrochloride or through temperature-induced denaturation. However, chemical and temperature methods that can be readily used with solid-phase nanopores can denature/destabilize protein nanopores and must be adjusted to account for this issue. Furthermore, raising sample temperature to disrupt secondary/tertiary structures can also increase translocation dynamics and the difficulty of detecting specific peptide sequences [90].

Most peptides lack a stable secondary structure and translocate without the need for a denaturation procedure and, thus, provide a valuable means to determine the basic steps in more complex protein translocations. Several protein nanopores have been used in peptide analysis studies based on their unique properties, including 3 Fragaceatoxin C (FraC) variants modified to form octameric (FraC-T1), heptameric (FraC-T2), and hexameric (FraC-T3) nanopores with distinct inner diameters (1.6, 1.1, and 0.84 nm, respectively) [89]. These FraC variants could discriminate peptides containing 22 to 4 amino acids, although the authors suggest that smaller peptides could be distinguished by further modifications, and discriminated 2 octamer peptides that differed by 44 Da because of a single N-terminal amino acid difference.

### Immunoassay-based peptide detection and quantification

Immunoassays have been used to analyze peptides in clinical samples for more than 50 years, but while these antibody-dependent assays can often have good overall specificity for their target peptides, they may lack sensitivity or fail to distinguish minor sequence variants of biological significance. Major challenges when developing these assays primarily involve the identification of available antibodies with adequate affinity and specificity for a peptide target and their inability to quantify biomarker changes with precision.

Western blot analyses have been extensively used to detect target protein and peptide in complex biological samples [91] because they can fractionate an array of peptides in a complex sample according to their intrinsic size and charge properties before target detection to reduce assay background noise. Material loss during protein/peptide transfer to a detection membrane can, however, reduce detection sensitivities. This can be a considerable problem for peptides and small proteins that can more readily pass through the membrane during transfer than polypeptides and can exhibit weaker membrane binding and greater losses during subsequent immunoassay procedures. Several approaches have been used to address such protein/peptide transfer problems, including vacuum and electrophoretic transfer methods that offer greater consistency and control. Size fractionation, transfer, and detection efficiencies are also influenced by the limits imposed by electrophoretic gel pore size, which involves trade-offs between resolution and loading capacity, as well as the affinity of the specific antibody, which can be limited by peptide composition and length. Western blot analysis are also labor-intensive, semiquantitative, and have a limit of detection of approximately 50 to 100 pg (~25 to 50 fM) [92] and are thus best suited to detect relative changes or de novo appearance of diagnostic biomarkers.

**Fig. 4.**
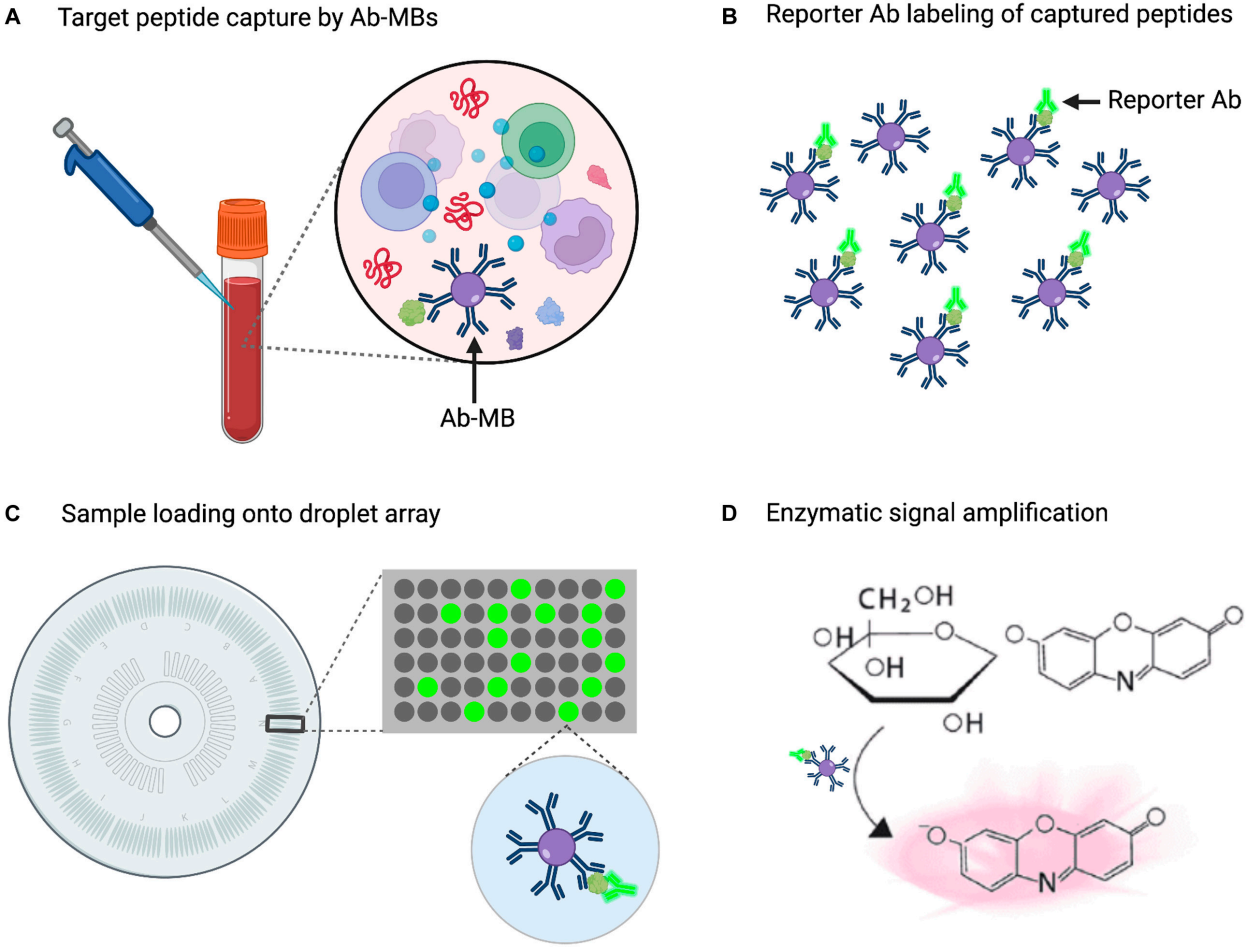
Digital ELISA platform. (A) Antibody-conjugated paramagnetic beads (Ab-MBs) are used to capture single-target biomarker molecules. (B) Bead-bound biomarkers are labeled with a reporter antibody. (C) Beads are then distributed on a microwell array that can capture a single bead per well. (D) Finally, the frequency of positive wells is determined by detecting a fluorophore-tagged reporter antibody or fluorescent activation of a reporter substrate by an enzyme-tagged reporter antibody.

Enzyme-linked immunosorbent assays (ELISAs) are also widely used to measure target peptide levels in body fluids for clinical diagnoses [93]. Sensitive immunoassays can detect proteins or peptides at concentrations ≥ 1 pM, but most proteins of importance in cancer, neurological disorders, and the early stages of infection are thought to circulate at 0.1 fM to 1 pM concentrations. A digital ELISA method that is approximately 1000-fold more sensitive than conventional ELISA was developed to address this gap (Fig. 4) [94]. In this method, antibody-conjugated MBs are employed to capture single protein/peptide biomarkers, then labeled with fluorophore-conjugated detection antibodies, dispensed into microarray wells capable of containing a single MB, and assessed for the presence or absence of a target-specific fluorescent signal. A commercial application of this approach is shown to detect 3 peptides biomarkers associated with neurological injury—neurofilament light chain, tau, and Aβ—in plasma and serum [95]. A subsequent traumatic brain injury study [96] found that serum neurofilament light chain levels measured by digital ELISA distinguished patients with traumatic brain injury and controls with much better overall sensitivity and specificity than standard ELISA (92% and 89% versus 31% and 100%), and the diagnostic performance difference was even larger when these methods were used to analyze serum tau (85% and 90% versus 7.7% and 100%). Digital immunoassays thus have potential utility in clinical applications that analyze low-abundance biomarkers but have long sample incubation times, low multiplex potential, and other features that limit their feasibility for routine use. A “pre-equilibrium digital ELISA” approach that can shorten standard ELISA incubation times up to 10-fold has been proposed to address the first of these issues [97]. In this modified digital ELISA method, rapid termination of the affinity binding step is used to ensure that MBs contain ≤1 immune complex over a wide range of target concentrations (10 fM to 1 nM).

### NP-based detection, quantification, and imaging strategies

**Fig. 5.**
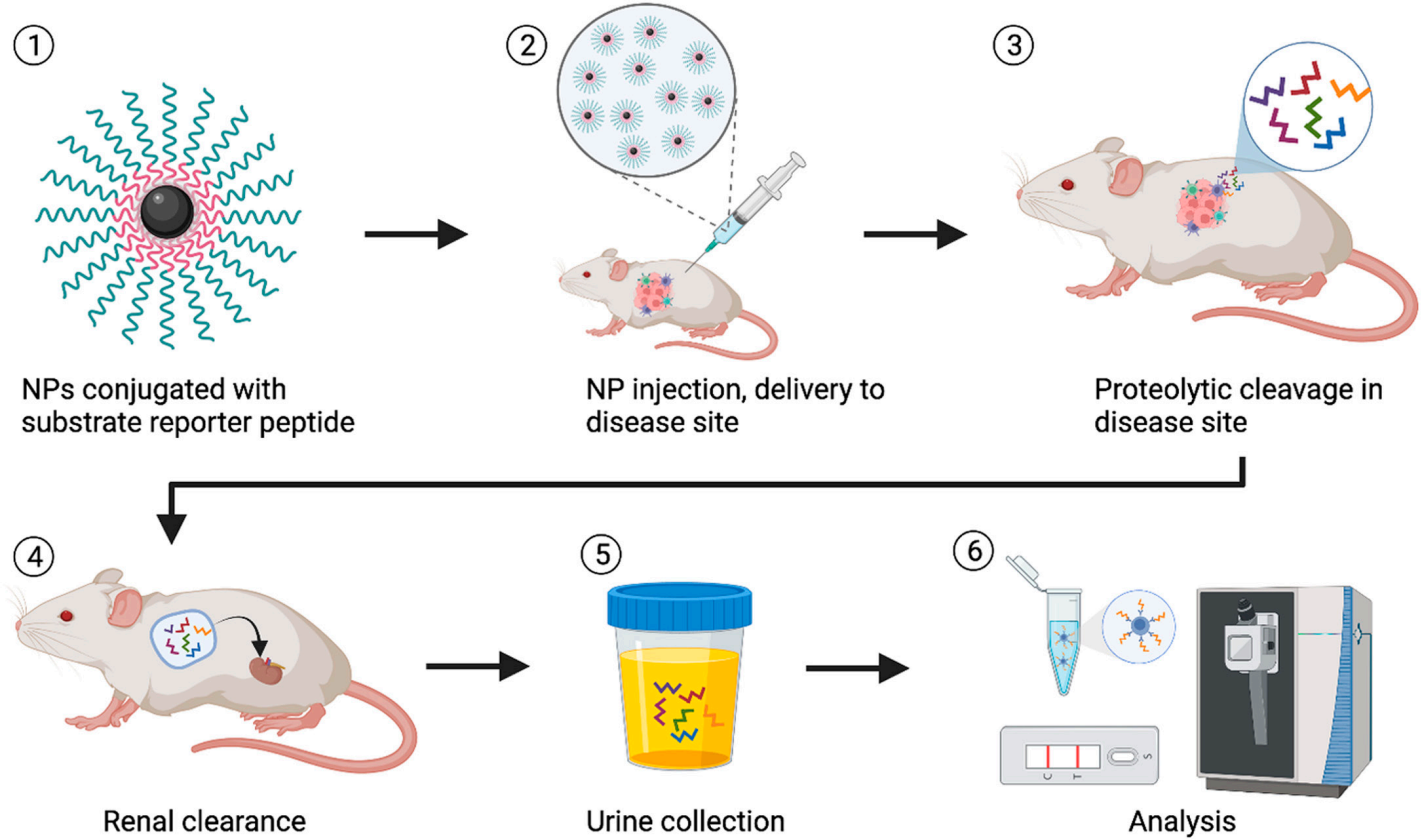
Schematic of the design and application of a synthetic NP–biomarker substrate system. Step 1: NPs are synthesized and conjugated with tagged protease substrates to detect altered protease activity in a target tissue. Step2: NPs are injected into a vein or into subcutaneous tissue and accumulate in the target tissue. Step 3: Protease activities at the target site cleave the synthetic biomarker substrates. Step 4: Cleaved substrates enter the circulation by tissue perfusion and are then rapidly removed by renal clearance. Step 5: Urine samples are collected to measure substrate release. Step 6: ELISA, MS, or other sensitive biomarker detection methods are used to measure biomarker release to diagnose disease or monitor it change in response to treatment.

Nanomaterial and nanotechnology applications analyzed by sensitive new instruments can accurately and noninvasively quantify biological processes. NPs conjugated with factors that have specific cell or tissue affinities can function as rapid, sensitive, and cost-effective targeted imaging/contrast agents, therapeutics, and diagnostics, and these activities can often be combined into a single NP for multiplexed analyses [98]. For example, one promising noninvasive diagnostic approach to detect or monitor altered activity associated with specific disease or its pathology is to administer an NP modified with a mass-tagged substrate for an enzyme of interest and evaluate the subsequent accumulation of the processed product in urine as a biomarker of a disease or its stage. A version of this approach using untargeted NPs conjugated with an array of protease substrates found that they passively accumulated in targeted tissues via increased disease-associated vascular permeability and detected altered protease activities associated with mouse models of liver fibrosis and colorectal cancer (Fig. 5) [99]. Similar methods therefore have the potential to improve early detection of cancer [100] and other diseases [101] through noninvasive measurement of enzyme activities that exhibit specific correlations with these pathologies. Variants of this approach have been applied for ultrasensitive detection of colorectal cancer [102], thrombosis [103], inflammation [103], and epithelial tumors and ovarian cancer [104].

NP applications for medical and preclinical imaging are a rapidly developing area in early diagnosis and can permit real-time and longitudinal visualization of physiologic and pathologic disease features [105]. NPs conjugated with affinity ligands (e.g., monoclonal antibodies, peptides, or small molecules) that recognize selected biomarkers can target cells and tissues with high specificity and affinity and can exhibit robust biodistribution, half-lives in circulation, tissue permeability, cell specificity and uptake, and image contrast properties. Peptide-functionalized NPs have been extensively used for early cancer diagnosis in animal models. For example, peptide-functionalized NPs have been used to image MDA-MB-231 xenograft-bearing nude mice [106]. Gold NPs conjugated with the α-melanocyte-stimulating peptide hormone and radiolabeled with ^64^Cu have been used for positron emission tomography imaging for early tumor detection in a mouse model of melanoma [107]. Similarly, gold NPs conjugated with a cyclic Arg-Gly-Asp (RGD) peptide that interacts with integrin α_v_β_3_ has been used to detect α_v_β_3_ integrin overexpression on colorectal cancer cells [108], although these studies were performed in vitro. However, with the exception of iron oxide NPs, NPs have not been incorporated into clinical diagnostic applications. This is primarily due to difficulties associated with achieving acceptable pharmacokinetic properties and reproducible uniformity of these NPs, as well as concerns about their toxicity, degradation, and elimination.

## Interest in Peptides as Biomarkers

Many diseases are associated with abnormal proteolysis. For example, perturbation of proteolytic degradation in neurons has been implicated in AD pathogenesis [109], while urine peptidomics has detected degradome signatures associated with specific renal, metabolic, autoimmune, cardiovascular, and malignant diseases [110]. Peptides produced by altered protease activities can thus have value as diagnostic biomarkers or indicate potential therapeutic targets. This has led to growing interest in identifying peptide biomarkers for use in new applications in diagnostic, predictive, preventive, and personalized medicine. This section will briefly discuss 3 major types of peptides biomarkers, specific examples of each, and some potential clinical applications.

### Enzyme substates

Multiple enzymes, including proteases, play essential roles in modulating signaling and immune responses, and the dysregulation or dysfunction of such enzymes is a major contributing factor in multiple diseases [111]. Peptides are the immediate downstream products of many biologically important enzymes and can thus often serve as specific and readily detectable surrogates of their activity. For example, Aβ production correlates with β-secretase expression, which may serve as a diagnostic marker for AD [112]. Recent studies have also shown that transglutaminases TG1, TG2, and FXIII-A present in osteoclasts regulate osteoclastogenesis to influence normal and pathogenic bone remodeling and that biotinylated “Hitomi peptides” reflect TG activity and can be used to specifically detect distinct TG functions [113]. Finally, a synthetic peptide derived from β-casein is reported to permit rapid, selective, and efficient monitoring of Golgi-enriched fraction case kinase expressed in lactating mammary glands, as it is not cleaved by ubiquitously expressed casein kinase 1 and 2 activity [114].

### Bioactive peptides

Bioactive peptides (e.g., cytokines, peptide hormones, and neuropeptides) play key roles in modulating disease development and pathology and may thus serve as useful disease biomarkers. For example, elevated serum levels of the cardiac hormone NT-proBNP detected in individuals with Anderson–Fabry disease—a genetic disorder that predisposes carriers to progressive renal, cardiac, and cerebrovascular disease—are associated with abnormal electrocardiographic findings of early cardiac involvement [115]. NT-proBNP measurement might therefore assist in decisions on the timing of enzyme replacement therapy used to delay or reverse adverse cardiac remodeling in Anderson–Fabry disease. Similarly, another peptide (BNP) derived from proBNP and 2 other members of the NP family [atrial NP (ANP) and C-type NP (CNP)] regulate sodium and water excretion [116]. Saliva levels of 3 antimicrobial peptides (HNP 1-3, LL-37, and S100) derived from epithelial and neutrophils are also elevated in patients with Behcet’s disease, a chronic inflammatory disease associated with systemic vascular inflammation [117], and HNP 1-3 and LL-37 levels exhibited an association with disease severity, perhaps reflecting the elevated level of enhanced local and systemic innate immune responses in Behcet’s disease [118].

### New insights into protein and peptide modifications

Protein and peptide PTMs are usually not evaluated when these factors are evaluated as biomarkers, but these PTMs may contain important biological information that may reflect disease states because they can modify or regulate protein and peptide activity and turnover, as well as affect interactions with other peptides or proteins that can exert substantial effects on pathogenic states in many diseases [119]. For example, amyloid plaques associated with AD contain multiple PTMs, including extensive N-terminal degradation and cross-linkages in stable Aβ aggregates found in these plaques, which could reflect or modulate disease progression [120]. Similarly, tau PTM profiles have been shown to indicate changes that occur in a sequential manner and reflect disease progression, as well as suggest that different intervention approaches may be required at different disease stages [121]. Plasma levels of B-type natriuretic peptide can also serve as a biological marker to differentiate cardioembolic stroke from small- and large-vessel disease and other ischemic stroke subtypes [122].

## Peptide Biomarker Applications in Diseases

There is a large and growing need for biomarkers to diagnose specific chronic and infectious diseases and evaluate their pathophysiologic states. Peptidomics has been applied to discover novel peptide biomarkers that can diagnose early disease, guide treatment decisions, and evaluate treatment response and disease prognosis. Several peptide-based diagnostic biomarker assays are now commercially available or under evaluation in clinical validation studies. This section will discuss peptide biomarkers currently used in clinical assays for diseases associated with 4 major disease categories or that have recently been identified as potential biomarkers for these diseases.

### Neurodegenerative diseases

Several biomarker peptides are associated with AD and PD, major progressive neurodegenerative conditions that affect the structure and function of the CNS or peripheral nervous system. AD is characterized by CNS accumulation of extracellular plaques primarily composed of Aβ produced by the cleavage of amyloid precursor protein (APP) via β-secretase (BACE1) and γ-secretase activity [123]. This process produces both soluble Aβ40 and insoluble Aβ42 that contains 2 additional C-terminal residues and can oligomerize to produce Aβ plaques [124]. The first discernible AD pathology is Aβ42 accumulation in extracellular plaques, followed by synaptic dysfunction and increased phosphorylation and secretion of tau, a microtubule-binding axonal protein highly expressed in cortical neurons [125]. AD biomarkers have primarily been analyzed in blood or cerebrospinal fluid (CSF) [126,127], although CSF may permit more sensitive analyses because it directly contacts CNS tissue and may thus more accurately reflect CNS changes. Studies have consistently demonstrated that CSF Aβ42 and total and phosphorylated tau (T-tau and P-tau) levels can function as diagnostic biomarkers for AD diagnosis. Aβ40 and Aβ42 are the most widely studied Aβ peptides. Reduced CSF Aβ42 expression has been reported to differentiate individuals with AD from age-matched controls without cognitive impairment and to also distinguish AD-related mild cognitive impairment and stable mild cognitive impairment cases [128]. Furthermore, in a cohort of individuals at risk for an autosomal dominant AD mutation, substantial differences in the carrier and noncarrier levels of CSF and plasma Aβ42 and CSF T-tau were detected at 10, 15, and 15 years before their expected age of symptom onset based on their family history [129], indicating their efficacy as earlier biomarkers of AD onset. Aβ peptide ratios are also used as biomarkers, including changes in plasma or CSF Aβ42/Aβ40, Aβ42/Aβ38, Aβ42/Aβ43, Aβ42/APP669-711, Aβ42/T-tau, or Aβ42/P-tau ratios [130]. Notably, the Aβ42/Aβ40 ratio is characteristic of prodromal and symptomatic AD and could aid in distinguishing dementia associated with AD, PD, and Lewy bodies in their prodromal stages [130]. Similarly, other endogenous peptides are reported to function as candidate biomarkers for other progressive neurodegenerative diseases. For example, CSF levels of a pleiotrophin peptide (amino acids 151 to 166) were found to progressively increase with severity of cognitive impairment in individuals with AD, mild cognitive impairment, and subjective cognitive decline, but not in healthy controls with no evidence of cognitive impairment or individuals with PD or progressive supranuclear palsy [131].

### Heart failure

HF is a major public health concern because of its growing prevalence, poor prognosis, and high health care costs, and new accurate early diagnosis methods are needed to limit pathology and improve patient outcomes. Members of the NP family (ANP, BNP, and CNP) regulate sodium and water excretion, and ANP, BNP, and NT-proBNP can act as biomarkers of hemodynamic stress [116]. BNP and its precursor NT-proBNP are widely used as diagnostic biomarkers for HF and cardiac dysfunction, with one systematic review indicating that blood NT-proBNP levels have 92% overall sensitivity and 88% specificity for HF diagnosis [132]. However, the utility of NPs as biomarkers when evaluating prognosis or response to therapy is less clear [133]. BNP and NT-proBNP also have value in ruling out acute heart failure (AHF) in emergency department patients exhibiting shortness of breath of unknown cause because AHF is a common preliminary diagnosis for hospitalized emergency department patients, but effective AHF diagnosis may also require lung ultrasound and echocardiographic evaluations [134].

### Infectious disease

Relatively few studies have examined the use of pathogen-derived peptides as diagnostic biomarkers for specific infectious diseases. Most clinical assays that use pathogen-derived peptides use them as targets to capture pathogen-specific antibodies in standard immunoassays, which cannot distinguish previous pathogen exposure from current infection. However, direct detection of pathogen-derived proteins can be problematic, because these proteins can be masked by interactions with abundant host proteins or through degradation by host protease activities. This can make it difficult to detect them in diagnostic specimens, particularly early in infection and following treatment intervention. It can also be difficult to distinguish closely related pathogens because of the sequence conservation of their major proteins.

Proteomic assays can address most of these issues because protease digestion of a specimen during its processing for proteomic analysis would disrupt inhibitory protein interactions, because LC-MS can resolve low-abundance targets in complex mixtures, and because MS^2^ analysis can distinguish species-specific peptides that differ at a single amino acid position. Most diagnostic proteomic methods analyze secondary cultures of clinical isolates instead of direct clinical specimens and match raw MALDI-TOF MS spectra that indicate relative ion intensity to reference spectral libraries, instead of identifying species-specific peptides. This makes these approaches slow and subject to variation because of culture effects and strain differences, although characteristic MALDI-TOF MS spectra have been employed to identify specific microbial pathogens (e.g., mycobacteria and *Candida* species) with high sensitivity and specificity [135–137]. However, MS has also been used to quantify serum levels of virulence factors expressed by *Mycobacterium tuberculosis* by identifying species-specific peptides derived from these proteins to rapidly and accurately diagnose tuberculosis [138,139]. This can have advantages over culture or polymerase chain reaction analysis of respiratory or tissue biopsy samples that are conventionally used for this purpose but which can yield false-positive results, particularly in patient population at increased risk for worse outcomes.

### Cancer

Peptide biomarkers have been reported for a variety of cancers. Serum and tissue oxytocin levels are substantially elevated in patients with prostate cancer and have been proposed as a biomarker for prostate cancer diagnosis and progression [140]. Serum proteomic profiling of patients with breast cancer using a separation protocol in which peptides bound to a carrier protein were isolated by affinity chromatography by MBs and subjected to MALDI-TOF MS analysis has also identified 3 peptide biomarkers (FGA 605-629, ITIH4 347-356, and APOA2 43-52) for breast cancer diagnosis and prognosis [141]. Similarly, a high-throughput MALDI-TOF MS workflow has also been used to identify several serum biomarker peptides that can differentiate patients with stage I ovarian cancer from age-matched disease-free controls [142], which could markedly improve 5-year survival rates for this cancer by increasing early diagnosis and intervention.

Peptide markers have also been identified that can predict or monitor cancer treatment responses. For example, a nanoporous silica chip coupled to MALDI-TOF MS method identified 3 plasma peptides that could discriminate the treatment response of patients with rectal cancer (responders versus nonresponders) to preoperative chemoradiotherapy with 91% sensitivity and 76% specificity, for an overall 86% accuracy [143].

## Conclusion and Perspective

Mounting evidence indicates that peptidomic information can be valuable for diagnostic and prognostic evaluation of chronic, malignant, and infectious disease conditions and their associated pathologies. Methodologic, bioinformatic, and analytic advances have addressed challenges for accurate detection and quantification of disease biomarkers present at a low concentration in complex specimens to enhance their feasibility for use in clinical applications. MS studies have revealed numerous peptide biomarkers that can distinguish disease and control groups, but substantial effort is still required to validate the clinical utility of these findings and transfer them to applications that can be feasibly used in a clinical laboratory setting. MS remains the most accepted means for peptide detection and quantification but require infrastructure and expertise that limit its use to well-equipped clinical laboratories. Nanopore systems hold promise as an inexpensive alternative approach but lack the separative and target discrimination capabilities of MS, and further instrument and software development is required to facilitate the design, production, and validation of nanopore-based clinical applications. New assays that utilize NPs for targeted delivery of synthetic biomarker substrates also appear to hold substantial promise as a minimally invasive means of evaluating biological processes that would otherwise require analysis of a tissue biopsy. However, clinical adoption of this in vivo approach will require additional studies to address regulatory concerns that do not apply to in vitro diagnostics. Maturation of these newer technologies should permit rapid, sensitive, and reliable peptide analyses to be performed using inexpensive systems that require less infrastructure and technical expertise, which should broaden access to peptide biomarker clinical applications.
